# The UHRF1 protein is a key regulator of retrotransposable elements and innate immune response to viral RNA in human cells

**DOI:** 10.1080/15592294.2023.2216005

**Published:** 2023-05-29

**Authors:** RE Irwin, C Scullion, SJ Thursby, M Sun, A Thakur, L Hilman, B Callaghan, PD Thompson, DJ McKenna, SB Rothbart, Guoliang Xu, CP Walsh

**Affiliations:** a Biomedical Sciences, Ulster University, Coleraine, UK; b Precision Nanosystems Inc, Vancouver, BC, Canada; c State Key Laboratory of Molecular Biology, Shanghai Institutes of Biological Sciences, Shanghai, China; d Cellular and Molecular Medicine Program, Division of Oncology, Johns Hopkins School of Medicine, St., Baltimore, MD, USA; e Department of Epigenetics, Van Andel Research Institute, Grand Rapids, MI, USA; f Nutrition Innovation Centre for Food and Health, Biomedical Sciences, Ulster University, Coleraine, UK

**Keywords:** Epigenetics, DNA methylation, UHRF1, Retrotransposons, Virus

## Abstract

While epigenetic mechanisms such as DNA methylation and histone modification are known to be important for gene suppression, relatively little is still understood about the interplay between these systems. The UHRF1 protein can interact with both DNA methylation and repressive chromatin marks, but its primary function in humans has been unclear. To determine what that was, we first established stable UHRF1 knockdowns (KD) in normal, immortalized human fibroblasts using targeting shRNA, since CRISPR knockouts (KO) were lethal. Although these showed a loss of DNA methylation across the whole genome, transcriptional changes were dominated by the activation of genes involved in innate immune signalling, consistent with the presence of viral RNA from retrotransposable elements (REs). We confirmed using mechanistic approaches that 1) REs were demethylated and transcriptionally activated; 2) this was accompanied by activation of interferons and interferon-stimulated genes and 3) the pathway was conserved across other adult cell types. Restoring UHRF1 in either transient or stable KD systems could abrogate RE reactivation and the interferon response. Notably, UHRF1 itself could also re-impose RE suppression independent of DNA methylation, but not if the protein contained point mutations affecting histone 3 with trimethylated lysine 9 (H3K9me3) binding. Our results therefore show for the first time that UHRF1 can act as a key regulator of retrotransposon silencing independent of DNA methylation.

## Introduction

DNA methylation is known to play an important role in mice in maintaining suppression at many genes which are transcriptionally inactivated during development and differentiation [1], such as those on the inactive X chromosome [2], silent alleles of imprinted genes [3], inactive olfactory receptor genes [4], some protocadherins [5], and certain germline genes [6]. These roles have largely been established by introducing mutations or deletions in the genes encoding the DNA methyltransferases (DNMTs), either in the whole embryo or in specific tissues such as the brain or germ line. DNA methylation has also been known for some time to be important for suppression of retrotransposable elements (REs) in mice, as hypomorphic mutations in the maintenance methyltransferase DNMT1 result in widespread de-repression of Intracisternal A Particles (*IAP*), a young and mobile class of RE specific to rodents [7]. Less is known about the transcriptional response to loss of DNA methylation in human, where developmental models are lacking. Studies there have been hampered by a strong cell-autonomous DNA damage response which occurs even in undifferentiated cells lacking DNMT1, and acute loss of the enzyme results in cell death within a few cell generations through triggering a DNA damage response [8–10].

Recent studies have shown that treatment of human cancer cell lines with pan-DNMT inhibitors (DNMTi) such as 5-aza-2’-deoxycytidine (5-AZA-CdR) led to demethylation and transcriptional up-regulation of endogenous retroviruses (ERV) [11–13]. ERV are a type of Class I transposable element which transpose using a copy-and-paste mechanism going through an RNA intermediate (i.e., retrotransposition), whereas Class II elements use cut-and-paste instead. Double-stranded RNA (dsRNA) generated by ERV in the cytoplasm has been associated with a recognition pathway implicating dsRNA sensors DDX58 (RIG1) and MDA5 (IFIH1), which triggered IRF7 signalling through the mitochondrial protein MAVS. IRF7 translocated to the nucleus and up-regulated interferons (IFN) and interferon-stimulated genes (ISG) which include dsRNA sensors and other upstream components in a feedback loop, triggering an innate immune response including presentation of peptides produced from the cancer/testis antigen (CTA) genes at the surface and cell-cell signalling [11,12]. Association of the CTAs with immune response to viral infection has also been reported [13,14]. The extent to which these effects are due to loss of DNA methylation only, or to secondary effects of the inhibitors is currently unclear though, since the effects have not been fully characterized in cells carrying DNMT1 mutations [11,15] and 5-AZA-CdR is known to affect levels of the histone methyltransferase G9a [16].

While the effects of DNMTi in humans and studies using mouse mutants have implicated DNA methylation in RE suppression, other mechanisms are also at work to ensure transcriptional suppression and avoid genomic disruption during periods of DNA hypomethylation in germ and stem cells, principally histone 3 lysine 9 trimethylation [17–19]. Consistent with this, loss of H3K9me3 leads to up-regulation of REs and RE-neighbouring genes in mouse stem cells [20]. Recent work in human leukaemia has also shown that SETDB1, a H3K9 methyltransferase, was required for repression of both long terminal repeat (LTR)-containing REs such as ERV, and non-LTR REs such as the long interspersed nuclear elements (LINEs) [21]. However H3K9me3 levels decrease in differentiated human cells, where DNA methylation is thought to take over as the primary suppressive mechanism [22,23].

Mutations in the Ubiquitin-like with PHD and ring finger domains 1 (*Uhrf1*) gene (aka *Np95*) were initially characterized as phenocopying loss of DNMT1 in mouse and resulted in widespread hypomethylation of the genome including RE such as *IAP* and *LINE-1* [24,25]. In cells lacking UHRF1 the DNMT1 protein did not localize correctly to the nucleus and the paired tandem tudor (TTD)- plant homeodomain (PHD) region of UHRF1 has been proposed to allow interaction with chromatin even during mitosis by binding to H3K9me3 [26,27]. Reports regarding the role of UHRF1 and more specifically H3K9me3 binding in DNA methylation have varied. Mutations in the TTD-PHD region that affect H3K9me3 binding by UHRF1 have been shown in human to decrease DNA methylation at ribosomal DNA repeats in HeLa cells [26], but effects on single-copy genes and REs were not investigated. In mouse, mutations in the same region gave only a mild effect with a 10% decrease in DNA methylation [28]. Other studies in mouse embryos suggested that *UHRF1* mutations caused widespread loss of methylation, but that it only played a minor role in RE suppression [29]. Recent work has nevertheless suggested that UHRF1 is important for targeting DNMT1 to H3K9me3-enriched REs in mouse embryonic stem (ES) cells [30]. In zebrafish, mutations in the zebrafish homologue were reported to result in clear ERV de-repression in the developing embryo and activation of the innate immune system [31] as for DNMTi in human, but through both double-stranded DNA and dsRNA signalling, not just dsRNA [32]. Conversely, a recent report by the same group indicated that UHRF1 KO in mouse liver was not sufficient in itself to de-repress RE [33].

There is therefore a lack of clarity regarding the role of UHRF1, what the cellular response to loss of this important epigenetic regulator would be, what genes would be most affected, and the dependence, if any, of DNA methylation on the TTD-PHD domain. As complete ablation of UHRF1 caused cell death in mouse ES cells once differentiated [24,25], as well as in differentiated human cells (REI, MS, GLX, CPW data not shown), we generated a hypomorphic series using shRNA in a hTERT-immortalized normal fibroblast cell line, as before [34]. To circumvent cell death using CRISPR (data not shown – sgRNA listed in Table S8), we used an shRNA approach to encourage strong sustainable and viable of *UHRF1*. We report here that an unbiased genome-wide screen showed widespread loss of methylation across most regions, but the major transcriptional response was consistent with de-repression of transposable elements, and the cellular response to the appearance of dsRNA in the cytoplasm. Rescuing the cells with intact UHRF1 could restore RE repression and switch off the innate immune response. Blocking H3K9me3-mediated silencing via mutation of the binding pocket on UHRF1 prevented RE suppression, suggesting this is upstream of DNA methylation.

## Materials and methods

### Cell culture and transfections

The wild-type (hTERT1604) lung fibroblast cell line [35] and derivatives were cultured in 4.5 g/l glucose DMEM with 10% FBS and 2 × 1% NEAA (all Thermo-Fisher Scientific, Loughborough, UK). SK-MEL-28 cells were purchased from the European Collection of Cell Culture and were grown in 4.5 g/l glucose DMEM supplemented with 10% FBS. Stable depletion of UHRF1 in hTERT1604 (U5/U10/UH4 lines) used pGIPz Lentiviral shRNAmirs (Horizon/Dharmacon), see Table S1 for sequences, used according to the manufacturer’s instructions. Briefly, overlapping primers incorporating siRNA sequences to target UHRF1 were made and ligated into pGIPz. The vector was linearized using *Xho*I and *Mlu*I, then 1 µg transfected into wild-type (WT) cells using Lipofectamine 2000 (Thermo-Fisher Scientific) prior to selection in puromycin (Sigma-Aldrich, Dorset, UK) to isolate single colonies, which were then expanded; selection was removed 24 hours (24hrs) prior to any experimental analysis. Rescue cell lines (WT10/18, PHD1/4/10, TTD9) were generated by transfecting UH4 cells with pCMV plasmids containing full length UHRF1 cDNA which was either intact (WT) or contained functional mutations in either the PHD or TTD domains as previously described [27]; specifically TTD1 Y188A, PHD1/2/10 D334A/E335A. Individual colonies were selected in G418 (Sigma-Aldrich) and expanded as above. DNMT1 KD cell lines (d8, d10, d16) were generated as previously described [32].

For transient KD experiments, 1 × 10^5^ cells/well were seeded in 6-well plates prior to reverse transfection as previously described [36] using 100 nM ON-TARGETplus SMARTpool siRNA (Table S2) or scrambled control (SCR) (all ThermoFisher Scientific). Post-transfection, cells were cultured in complete medium to allow recovery, with extraction of RNA and DNA up to 28 days after addition of siRNA. For drug treatment (see Table S3) Ruxolitinib (Absource, München, Germany) was dissolved in DMSO and added to culture media at a final concentration of 2 µM; negative controls contained just DMSO. For analysis of dsRNA and dsDNA sensing pathways, cells were treated at a final concentration of 10 µg/ml Poly(I:C) or sonicated salmon sperm DNA in sterile (Agilent, Stockport, UK) for 72hrs, with fresh media and drug every 24hrs; the nucleic acids were dissolved in sterile phosphate-buffered saline (PBS), heated at 50°C and cooled on ice to achieve re-annealing into double strands prior to treatment. For the media transfer test, UH4 cells were seeded and grown for 72hrs, then media transferred onto the WT hTERT1604 cells, which were grown for another 72hrs.

### Immunohistochemical staining

Approximately 10,000 cells were seeded onto sterile glass slides and allowed to attach overnight. Cells were then fixed in 4% paraformaldehyde in PBS for 20 mins before being permeabilized with 0.1% Triton X-100 and pre-blocked with 1% BSA and 10% Normal Goat Serum (both Sigma-Aldrich) for 1 hr at 37°C. Slides were incubated with J2 primary antibody (Scicons, Szirák, Hungary, see Table S4 for antibodies) at 1:500 overnight at 4°C. The next day, slides were PBS-washed and incubated with anti-mouse IgG (H+L), F(ab’)2 Fragment (Alexa Fluor 488 Conjugate antibody (Abcam) at 1:500 for 1 hr at room temperature (RT) before PBS-washing and coverslip mounting on a slide using Dapi Eventbright Antifade Mountant (Thermo Fisher Scientific). Fluorescent images were taken with Zeiss Zen microscope (Oberkochen, Germany) using the 40× objective lens. Images were processed using Adobe Photoshop. Representative images from duplicate experiments.

### DNA analyses

Genomic DNA was extracted from cells growing in log phase, with each cell line done in triplicate, including one biological replicate. DNA preparation, bisulphite conversion and array hybridization were as previously described [36,37]. Briefly, DNA was isolated using the QIAmp DNA Blood Mini Kit (Qiagen, Crawley, UK), assessed for integrity and quality using a range of measures including agarose gel electrophoresis, UV absorbance and Quant-iT PicoGreen dsDNA assay (Thermo Fisher Scientific). Purified DNA was sent to Cambridge Biological Services, bisulphite converted using the EZ DNA Methylation kit (Zymo Research, California, USA) and run on the Infinium HumanMethylation450 BeadChip [38] and iScan.

For pyrosequencing, DNA (500 µg) was bisulphite-converted in-house as above, then PCR-amplified using the PyroMark PCR kit using Qiagen’s pyrosequencing primer assays or those designed in-house (see Table S5) via the PyroMark Assay Design Software 2.0 (Qiagen). Reaction conditions were: 95°C, 15 minutes (mins); followed by 45 cycles of 94°C, 30 seconds (secs); 56°C, 30secs and 72°C, 30secs; final elongation 72°C, 10 mins with products verified on agarose gels prior to pyrosequencing using the PyroMark Q24 (Qiagen).

### RNA analyses

RNA was extracted from cells growing in log phase using the RNEasy Mini Kit (Qiagen), including a DNase step. Complementary DNA (cDNA) was reverse transcribed in a reaction containing 250-500ng total RNA, 0.5uM dNTPs, 0.25 µg random primers (Roche, UK), 1× reverse transcriptase buffer and 200 U RevertAid reverse transcriptase in a total volume of 20 µl. Reaction conditions were as follows: 25°C, 10 mins; 42°C, 60 mins; 70°C, 10 mins. cDNA was stored at −80°C until use. Each RT-PCR reaction contained 1 µl cDNA from the above reaction, 1× buffer, 0.4 mM dNTPs, 1 µM primers (Table S6), MgCl_2_ concentration specific to the primers and 0.01 U Taq polymerase. PCR conditions were: 94°C, 3 mins; followed by cycles of 94°C, 30 secs; gene-specific annealing temperature for 1 min; 72°C, 1 min; final elongation 72°C, 5 mins. RT-qPCRs were performed using 1× LightCycler 480 SYBR Green I Master (Roche), 0.5 μM primers (Table S7) and 1 μl cDNA. Reactions were run on the LightCycler 480 II (Roche), with an initial incubation step of 95°C, 10 mins; followed by 50 cycles of 95°C, 10secs; 60°C, 10secs and 72°C, 10 secs. Expression was normalized to *HPRT*, and relative expression was determined using the ΔΔC_T_ method.

Array work was carried out as previously described [36,37]: briefly, total RNA was extracted from each cell line growing in log phase in triplicate, including at least one biological replicate, and was assessed for integrity and quantity using a SpectroStar (BMG Labtech, Aylesbury, UK) and bioanalyser (Agilent Technologies, Cheadle, UK) prior to sending to Cambridge Analytical Services for linear amplification using the Illumina TotalPrep RNA Amplification Kit (Life Technologies/Thermofisher, Paisley, UK) followed by hybridization to the HumanHT-12 v4 Expression BeadChip.

### Bioinformatics and statistical analysis

Output files in IDAT format were processed and bioinformatic analysis was carried out using the RnBeads [39] methylation analysis package (v1.0.0) as before [37]. To map CG sites showing highly reproducible changes (FDR <0.05) against the locations of RefSeq genes on the UCSC genome browser [40], we employed CandiMeth [41] on the Galaxy platform [42]. Absolute *β* levels were used to measure median methylation across genes of interest using CandiMeth, with further statistical analyses in Statistical Package for the Social Sciences software (SPSS) version 22.0 (SPSS UK Ltd).

Agilent arrays GSE93142 and GSE93135 from SuperSeries GSE93136 [15] were processed using the R package GEOquery (2.46.15), annotation package hgug4112a.db (3.8) and annotation table for Agilent -014,850 Whole Human Genome Microarray 4 × 44K G4112F (Probe Name version) from GEO to obtain log2 normalized fold changes (FC) per probe. Gene Ontology analysis through DAVID [43] was then computed using the top 500 genes with greater than 1.5 FC.

Statistical analysis for pyrosequencing and RT-qPCR data using Student’s paired t-test employed Microsoft Excel (Microsoft Office Professional Plus 2016). Experiments were carried out at least in triplicate and included at least one biological replicate in all cases except Fig. S1, one biological repeat only. Error bars on all graphs represent standard error of the mean (SEM), or in the case of HT12 array data, 95% confidence interval (CI), unless otherwise stated. Asterisks are used to represent probability scores as follows: *p < 0.05; **p < 0.01; ***p < 0.001or n.s. not significant.

### Protein analysis

Cells in log phase were harvested using protein extraction buffer (50 mM Tris – HCl, 150 mM NaCl, 1% Triton-X, 10% glycerol, 5 mM EDTA; all Sigma-Aldrich) and 0.5 µl protease inhibitor mix (Sigma-Aldrich). Western blotting was carried out essentially as before [36]: in brief, 30 μg protein was denatured at 70°C in the presence of 5 μl 4× LDS sample buffer and 2 μl 10× reducing agent (Invitrogen) in a total volume of 20 μl nuclease-free water (Qiagen). Proteins were separated by SDS-PAGE and electroblotted onto a nitrocellulose membrane (Thermo-Fisher Scientific), then blocked in 5% non-fat milk for either 1 h at RT or overnight at 4°C. Membranes were incubated with the primary antibody (Table S4) overnight at 4°C, followed by HRP-conjugated secondary antibody incubation at RT using ECL (Thermo-Fisher Scientific).

## Results

### Widespread DNA demethylation in cells depleted of UHRF1 is accompanied by a specific innate immune response

**Figure 1.**
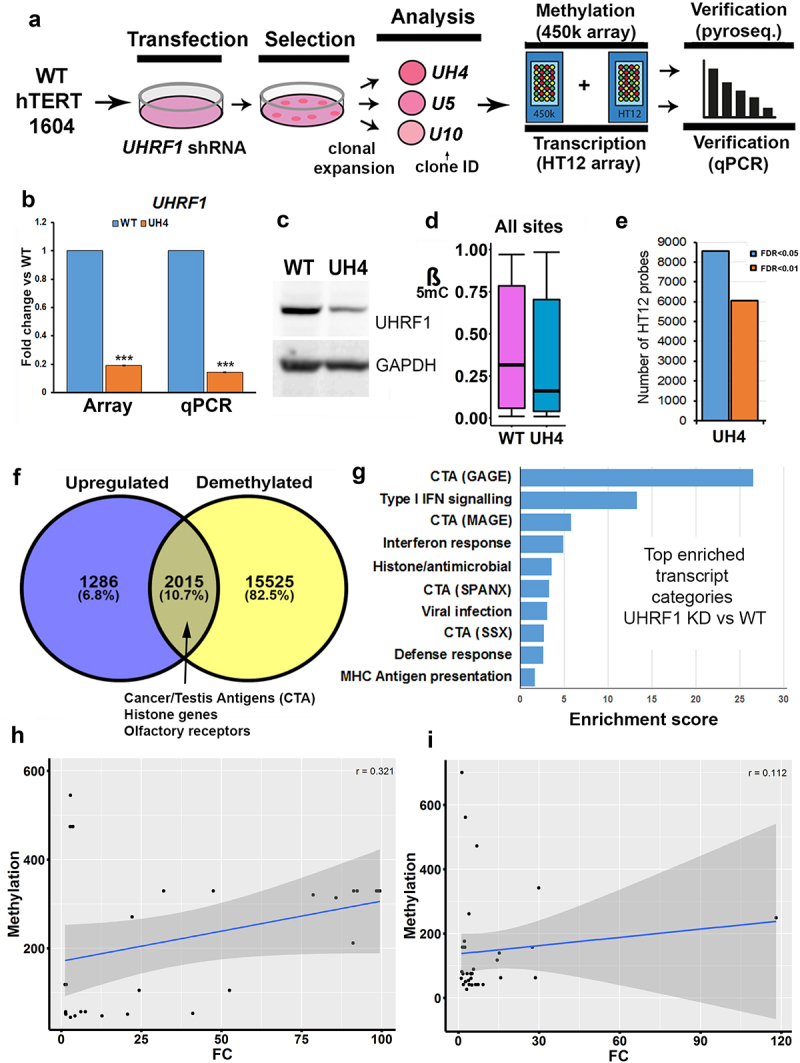
Loss of DNA methylation in UHRF1-deficient human fibroblast cells indirectly triggered a viral defence response.

Loss of function mutations in the human *UHRF1* gene introduced using CRISPR-Cas9 approaches failed to return any viable cell colonies on multiple attempts in different cell lines (REI, MS, GLX, CPW data not shown), in line with observations of cell death following differentiation of mouse ESC with *Uhrf1* mutations. We therefore used stably integrated shRNA to reduce rather than ablate UHRF1 in normal human fibroblasts which have been immortalized using hTERT (hTERT-1604) (Figure 1a). Two rounds of experiments were carried out using shRNA targeting either the main body (prefix U e.g., U5) or the 3’UTR (prefix UH e.g., UH4) of the gene. Results were indistinguishable; those for the index line UH4 are shown here as an example, results from other clones were similar and samples are shown in Figure S1. Initial screening was using reverse transcription-polymerase chain reaction (RT-PCR): cells showing depletion were further expanded and *UHRF1* mRNA levels checked by quantitative RT-PCR (RT-qPCR; Figures 1b, S1A) as well as checking protein levels by western blotting (Figures 1c, S1B). Lines showing depletion were further analysed using HT12 arrays for transcription, which verified low *UHRF1* levels (Figure 1b), as well as 450K array for DNA methylation. Median methylation levels, expressed as a β value between 1 (fully methylated) and 0 (no methylation) were significantly lower in UH4 than WT (Figure 1d, Fig. S1C). Examination of the transcription profile indicated that many probes showed significant differences between UH4 and WT using false discovery rates (FDR) as low as 0.01 (Figure 1e).

(a) Schematic overview: normal fibroblasts (hTERT1604) were stably transfected with shRNA, then single cells expanded and evaluated for genome-wide transcription (HT12) and methylation (450K) using microarrays: hits were verified using pyrosequencing and RT-qPCR. (b) Decreased *UHRF1* mRNA levels in the index UH4 line from HT12 array and RT-qPCR; error bars are 95% confidence interval and standard error of the mean, *** p < 0.001 Mann-Whitney U or T-test, respectively. RT-qPCR was performed in duplicate including one biological replicate from the clone with best siRNA KD to match HT12 array. Error bars in all experiments represent standard error of the mean (SEM). (c)Western blot showing levels of UHRF1 protein and GAPDH as a loading control. This was performed in triplicate, including at least one biological replicate. (d) Boxplot showing median and inter-quartile range of DNA methylation (β) values across all probes from the 450K array in parental (WT) and UHRF1-depleted (UH4) cells; difference between samples significant at p < 2.2×10^−16^ (Kruskal-Wallis test). (e) Number of probes on transcription (HT12) array showing significant deregulation at indicated false discovery rate (FDR) values for UHRF1-depleted (UH4) cells (f) Comparison of gene promoter regions showing >10% (0.1 β) demethylation and those showing transcription changes >1.2-fold. (g) Gene Ontology (GO) analysis on genes showing the most transcriptional de-repression, regardless of methylation. The top 10 categories from DAVID functional annotation clustering with enrichment scores are shown: all are related to an innate immune response to virus infection. The x-axis represents group enrichment score, the geometric mean (in –log scale) of the p-values of the individual subcategories. IFN, interferon; MHC, major histocompatibility complex; different subclasses of CTA are shown in brackets. (h) Logistic regression showing correlation between DNA methylation (methylation) and gene expression (FC) for cancer-testis antigens (r = 0.321) and (I) viral defence genes (r = 0.112).

We concentrated for our expression analysis on recently active or ‘young’ full-length retroviruses which will invoke a cellular viral response and on L1PBA, a rare example of a young, full-length L1. Primers for the specific young HERVs and L1 were taken from two recent publication demonstrating that they can be active in response to DNMTi [13,18]. The goal of our work here was to show transcription from full-length active retroviral elements which could invoke a viral defence response. DNA demethylation was widespread across the genome in UH4, with over half of all promoters (*n* = 17,540 yellow circle Figure 1f) showing significant decreases in β value (>0.1). Interestingly, when these were compared with genes showing up-regulation from the set of dysregulated transcripts the majority of genes (82.5%) showed demethylation but no de-repression. A relatively small percentage (10.7%) were both demethylated and upregulated, consistent with a direct role for DNA methylation in their suppression (Figure 1f). This included several gene categories known to be regulated by DNA methylation, such as Cancer/Testis antigen (CTA) genes and olfactory receptors (OR) [36]. Interestingly a third group of genes (6.8%) showed no demethylation but were nevertheless up-regulated (Figure 1f) suggesting an indirect response to the loss of DNA methylation. A total of 3352 genes were downregulated in UH4 with only 396 showing gain of methylation: these were related primarily to division and DNA replication (Supplementary Table S1).

To investigate transcriptional response more closely, we then carried out gene ontology (GO) analysis of all up-regulated transcripts using the DAVID clustering tool [43]. Top hits in this analysis (Figure 1g) included several sub-classes of CTA genes (GAGE SPANX, MAGE). The other enriched gene categories included Type I interferon (IFN) signalling, antiviral response and MHC antigen presentation (Figure 1g). In fact, all 10 of the top 10 categories are related to innate immune response to viral infection. For downregulated genes, these were associated with the GO terms for cell division and DNA replication. Methylation-independent regulation for viral defence genes was confirmed by a poor score on logistic regression (*r* = 0.112) (Figure 1h) in comparison with the CTA control (*r* = 0.321) (Figure 1i).

### Innate immune signalling is crucial to the cellular response following loss of UHRF1

**Figure 2.**
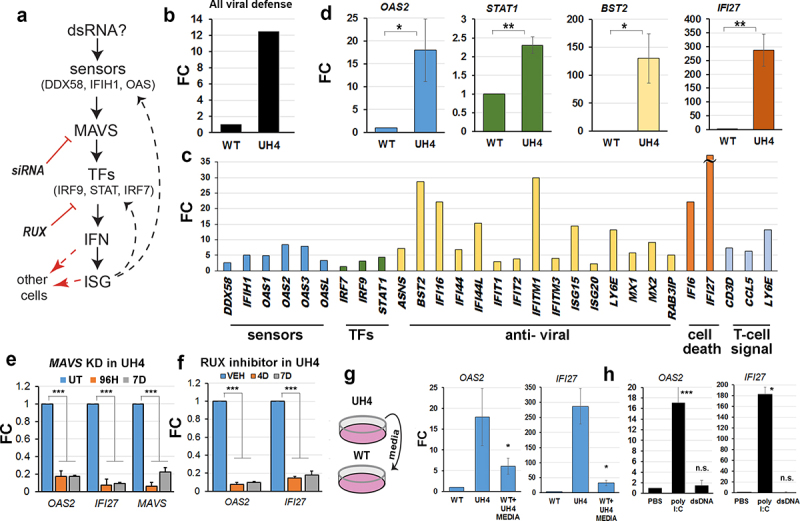
Interferons and interferon-stimulated genes (ISG) involved in dsRNA detection were crucial to the cellular response to loss of UHRF1.

Viral nucleic acids were detected in the cytoplasm by specific sensors, triggering a cascade which includes activation of ISG in the nucleus and secretion of signalling factors (Figure 2a) [44]. Average transcription among genes involved in viral response was clearly higher in UH4 cells compared to WT (Figure 2b). The profile of individual genes included in Figure 2C showed activation of components from several parts of the pathway, being greatest for ISG which are at the bottom of the cascade, including genes with anti-viral and cell death effects, and least marked for transcription factors (TFs) and sensors. Notably, three of the genes unique to our profile and not previously reported are linked to T-cell signalling (Figure 2c, right hand side). We verified sample genes from various parts of the pathway using RT-qPCR (Figure 2d), with results consistent in direction, though not always in magnitude, between the array and the RT-qPCR. There was also a poor correlation between methylation and transcription for the IFN and ISG genes (Figure 1i), suggesting the response is indirect.

(a) Model for possible pathway triggering ISG response in UH4 based on GO analysis and literature. Signalling from the dsRNA sensors would converge on the MAVS complex if dsRNA was detected, leading to release of transcription factors (TFs) which trigger upregulation of both interferons (IFN) and ISG. Many ISG are also part of the pathway forming a feed-forward loop (dashed arrows). Signalling to other cells can also occur (dashed red arrows). Inhibition of the pathway using siRNA against MAVS and the STAT inhibitor RUX are indicated. (b) Average fold change (FC) in UH4 versus WT cells (set to 1) for all viral defence genes determined using the HT12 transcription array. (c) Many components of the signalling pathway are upregulated on the transcriptional array: actual FC for *IFI27* was 118. (d) Verification of selected array targets from different parts of the pathway using RT-qPCR. RT-qPCR was performed at least four times with one biological replicate. Error bars in all experiments represent standard error of the mean (SEM). Significance was denoted as *p < 0.05; **p < 0.01; ***p < 0.001; n.s, non-significant, Student’s T-test. (e) An siRNA was used to KD MAVS in UH4 cells for the indicated period before assaying the named genes using RT-qPCR. Four independent siRNAs were used using SMARTpool approach. (f) UH4 cells were treated with the JAK/STAT inhibitor RUX for the indicated time before carrying out RT-qPCR on the named targets. (g) Schematic (left) of experiment where media which had been exposed to UH4 cells was transferred to plates containing WT cells (WT+UH4 media), before assaying transcription by RT-qPCR (right). (h) Exposure of WT cells to dsRNA (poly I:C), but not dsDNA, results in up-regulation of the same ISG as seen in UH4, measured here by RT-qPCR.Figure 2e-h: Experiments were performed in duplicate including one biological replicate. Error bars in all experiments represent standard error of the mean (SEM). Significance was denoted as *p < 0.05; **p < 0.01; ***p < 0.001; n.s, non-significant, Student’s T-test.

We tested this model mechanistically using several approaches. Inhibition of MAVS with siRNA in the UH4 cell line caused significant down-regulation of downstream ISG such as *IFI27* (Figure 2e). We also treated cells for 4–7 days with Ruxolitinib (RUX), a small-molecule inhibitor of JAK kinases which had a similar effect (Figure 2f). To test for cell-cell signalling, we transferred media from tissue plates containing UH4 cells to plates with WT cells (Figure 2g). This resulted in up-regulation of two highly responsive ISG, namely *OAS2* and *IFI27*. All the results above are consistent with an up-regulation of the dsRNA sensing pathway in the cells, presumably in response to the presence of dsRNA in the cytoplasm of UH4 cells (Figure 2a). Treatment of WT cells with polyI:C, a form of dsRNA, but not with dsDNA, caused up-regulation of the same genes as seen in the UH4 line, confirming that the transcriptional response is consistent with exposure to dsRNA (Figure 2h).

### The presence of dsRNA correlated with transcriptional de-repression and loss of DNA methylation at retrotransposable elements

**Figure 3.**
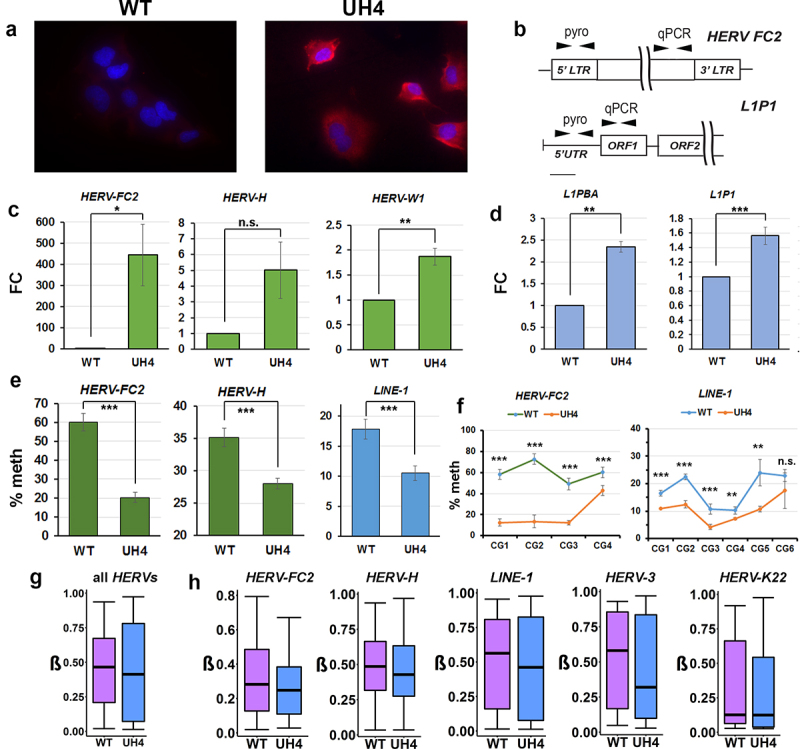
Retrotransposable elements were demethylated and transcriptionally activated by depletion of UHRF1.

Type I interferon response can be triggered in cells when dsRNA from invading virus or endogenous retrovirus is detected in the cytoplasm [11,12,21]. Staining of cells with the J2 monoclonal antibody is a sensitive and specific test for dsRNA [45] and gave a clear positive response in UH4, but not WT cells (Figure 3a). To test for de-repression of REs we designed RT-qPCR assays (Figure 3b) for family members activated in response to DNMTi [15,21], as transposable elements are not covered on the HT12 array. Members of several HERV families were transcriptionally up-regulated, including elements of the HERV-F (HERV-FC2), HERV-H (HERV-H) and HERV-W (HERV-W1) families (Figure 3c). As the FC was small, despite significant dsRNA signal from J2 staining (Figure 3a), we considered that other Class I RE might also be upregulated. LINE-1 elements are non-LTR REs which can stimulate an IFN response and are present at much higher copy number than HERV [21]. RT-qPCR was again consistent with up-regulation of some of these elements (*L1-PBA, L1P1*) in UH4 compared to WT controls (Figure 3b, d). Although small in magnitude (~2-fold), the absolute amount of dsRNA generated would be larger due to the greater copy number of the elements.

(a) WT and UH4 cells were stained with J2 monoclonal antibody (red), used for detection of viral dsRNA; nuclei were counterstained with DAPI (blue). (b) Locations of primers used to assay methylation (pyro) at the promoters and transcription (qPCR) relative to the retrotransposable elements (RE) indicated. LTR, long terminal repeat; UTR, untranslated region; ORF, open reading frame. (c) RT-qPCR for the indicated *HERV* elements showing fold-change over WT. (d) RT-qPCR for the *LINE-1* retrotransposable elements indicated. (e) Percentage DNA methylation (% meth) at the promoters of the indicated retrotransposons was determined using pyroassay. (f) Methylation across individual CG dinucleotides in the pyroassays indicated in E. Figure 3c-2f: Experiments were performed in at least triplicate including at least one biological replicate. Error bars in all experiments represent standard error of the mean (SEM). RT-qPCR for the indicated *HERV* or *LINE* elements showing fold-change over WT: *p < 0.05; **p < 0.01; ***p < 0.001; n.s, non-significant, Student’s T-test. (g) Methylation values (β) at all probes overlapping HERV elements from the 450K array, difference significant at p < 2.2×10^−16^ (Kruskal-Wallis test). (h) Methylation at the indicated retrotransposable elements using probes from the 450K array; LINE-1 difference significant at p < 2.2×10^−16^ (Kruskal-Wallis test), others n.s. but with lower probe numbers. Error bars indicate SEM (C-F) or 95% confidence interval (g, h).

Using pyrosequencing assays designed to cover multiple CG dinucleotides in the control regions (Figure 3b), we found consistent and significant demethylation of REs, including *HERV-FC2, HERV-H* and *LINE-1* (Figure 3e). Examination of individual CG confirmed significant demethylation across the entire region (Figure 3f). While the 450K array was not designed to assay repetitive elements, a substantial number of probes overlap with regions labelled as RE on the *RepeatMasker* track in UCSC. Using in-house scripts and our GALAXY workflow CandiMeth [41] we found median methylation across all REs in the genome to be significantly decreased (*p* < 2.2×10^−16^, Kruskal-Wallis test) in UH4 cells (Figure 3g). We also found evidence of substantial demethylation of several individual RE families (*HERV-FC2*, *HERV-H, LINE-1, HERV-3*), but not for all elements (*HERV-K22*) (Figure 3h). We also confirmed demethylation and up-regulation for RE and ISG for a number of other independently-derived clones from the two rounds of transfection (Figure S1 C-E). We confirmed small loss of DNA methylation levels globally for SINE elements using probes available on the 450k array (Figure S1F) however lack of activation of these elements (Figure S1G). Expression of *DNMT1* was unaltered upon UHRF1 KD, as confirmed by HT12 expression array, using *DNMT1* KD cell lines as control (Figure S1H). As UHRF1 KD resulted in widespread hypomethylation, we confirmed predominant loss of methylation for UH4 cells occurred at CGs in open sea regions, shelves and shores, respectively perhaps in keeping with RE activation (Figure S1I).

### A conserved interferon response follows RE demethylation in multiple cell types

**Figure 4.**
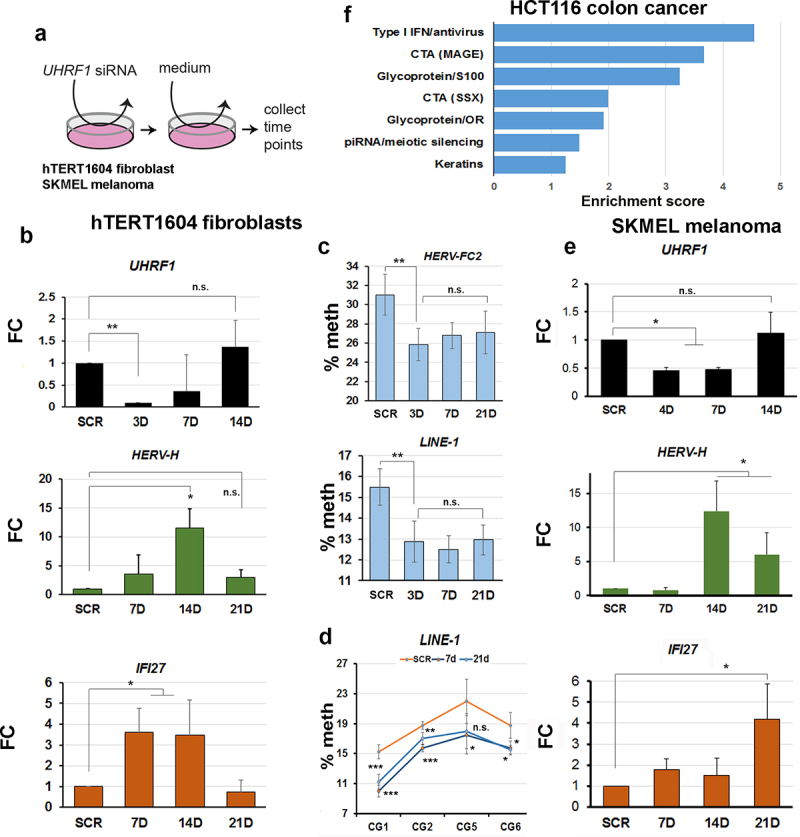
Demethylation of REs preceded reactivation and an interferon response in multiple cell types depleted of UHRF1.

To examine the timing of RE reactivation and innate immune response to loss of UHRF1, we carried out a ‘hit-and-run’ experiment (Figure 4a) where we exposed cells to siRNA against *UHRF1* for 48hrs, then switched the cells to normal medium without siRNA and allowed them to recover. *UHRF1* levels were effectively depleted to ~25% by 3D (Figure 4b), after which point they steadily recovered, reaching and even slightly exceeding levels seen in SCR by 14D. Consistent with observations in stable clones, *HERV-H* mRNA levels increased, starting already at 4D, and climbing steadily until 14D, at which point they started to decrease and were back at levels seen in SCR control by 21D (Figure 4b). The ISG gene *IFI27* showed comparable dynamics (Figure 4b), increasing from 7D and then decreasing to normal levels, or below, by 21D. RE showed loss of methylation at their promoter regions already at 3D (Figure 4c), consistent with this preceding activation. Interestingly, methylation showed only a modest gain (difference vs 3D not significant by T-test) during the recovery period and remained significantly lower than WT out to 21D, beyond the period during which transcription of the RE and ISG had already normalized (Figure 4c). This was also true for methylation levels at individual sites (Figure 4d 7D vs 21D not significant except CG2, *p* < 0.05).

(a) “hit-and-run” strategy was employed to establish timing of events: indicated cell types were exposed to small interfering RNA (siRNA) targeting *UHRF1*, or a scrambled control (SCR), for 48hrs, then fresh medium without siRNA added and cells allow to recover before sampling. Four independent siRNAs were used using SMARTpool approach. (b) RT-qPCR showing initial loss of *UHRF1* is followed by recovery to above initial levels by 14 days (14D). Levels of transcript for a representative ERV (*HERV-H*) and ISG (*IFI27*) are shown. FC, fold-change; error bars and statistics as above. (c) Average methylation levels at representative RE as determined by pyroassay; error bars are SD. Methylation recovery by 21 days (21D), when transcription is already repressed, is still not significant. (d) Differences at the most highly methylated individual CG sites for the LINE-1 assay compared to SCR, error bars are SD; 7D vs 21D n.s. except CG2, p < 0.05 (e) RT-qPCR analysis of SKMEL melanoma cells treated as in B. (f) GO analysis of genes showing transcriptional upregulation in HCT116 colon cancer cells following 90% KD of *UHRF1* (Cai et al, 2018). Enrichment scores etc as for Figure 1. Cells were analysed 7 days after adenoviral delivery of shRNA; raw data were obtained from GEO (GSE93136). Figure 4b-e: Experiments were performed in triplicate including one biological replicate. Error bars in all experiments represent standard error of the mean (SEM). Significance was denoted as *p < 0.05; **p < 0.01; ***p < 0.001; n.s, non-significant, Student’s T-test.

To determine if similar transcriptional responses would be seen in tumour cells, we performed an identical experiment in the SKMEL melanoma cell line, known to respond to DNMTi. Transient KD was less efficient, but *UHRF1* levels were depleted to ~50% by 7D, then rapidly recovered by 14D (Figure 4e). This was accompanied by activation of RE and ISG, peaking between 14D-21D, after which point transcription started to decrease again for the ERV, while the ISG was still up-regulated but more variable (Figure 4e). Additionally, we reanalysed a publicly-available dataset [15] where *UHRF1* was depleted in HCT116 colon cancer cells using adenovirus-mediated transfection of shRNA and where only a limited analysis of ISG by RT-qPCR had been reported. Using GO analysis of the RNA-seq data, we found enrichment for similar terms as in our UH4, including Type I interferon, CTA activation and interestingly, piRNA/meiotic silencing (Figure 4f). The sequencing depth in the dataset was not enough to enable a comprehensive RE expression analysis. Taken together with the results above, this confirmed that *UHRF1* depletion led to reproducible RE demethylation and de-repression in multiple cell types, evoking a strong innate immune response.

### Rescuing stable KD clones with UHRF1 can restore RE repression without re-establishing normal DNA methylation levels

**Figure 5.**
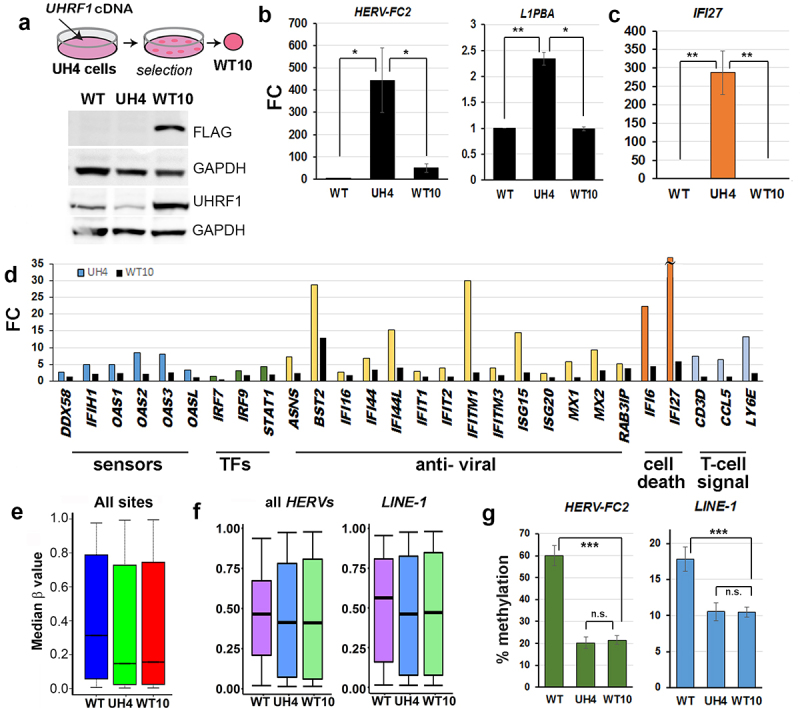
Rescuing cells with UHRF1 abrogated RE reactivation and interferon response without restoring DNA methylation.

The transient experiments above suggested, importantly, that RE repression could be re-established without full remethylation (Figure 4c, d). To confirm this in a more stable system, we rescued *UHRF1* expression in UH4 cells by transfecting them with full-length, FLAG-tagged cDNA lacking the 3’UTR which is targeted by the shRNA in UH4 (Figure 5a). Western blotting confirmed the presence of the FLAG-tagged protein in rescues, termed WT10 (Figure 5a). WT10 cells showed clear restoration of repression (Figure 5b) at HERVs (*HERV-FC2*) and LINE-1 elements (*L1PBA*). Reinforcing this, normalization of ISG levels was also seen (Figure 5c). Analysis of transcription by HT12 array confirmed widespread shut-down of the innate immune response, with genes from most components of the pathway returning to normal or near-normal levels (Figure 5d, black columns), with *BST2* as an exception. In stark contrast, median methylation levels overall in WT10 were indistinguishable from UH4 (Figure 5e), with no increase in methylation (β) in WT10 vs. UH4 at HERV or LINE-1 elements, as confirmed by both array and pyroassay analysis (Figure 5f, G). These results, taken together with the transient experiments in Figure 3 , indicated that UHRF1 can restore RE repression even when DNA methylation levels cannot be fully re-established.

(a) Schematic (top) showing rescue strategy: a plasmid containing a selectable marker and a full-length*UHRF1* cDNA lacking the 3’UTR targeted by the shRNA was transfected into UH4 cells and resistant colonies expanded. Western blot (bottom) detected the presence of the full-length FLAG-tagged UHRF1 and UHRF1 in index daughter cell line WT10 (b) RT-qPCR of the indicated retrotransposable elements showing repression in the WT10 cell line derived from UH4 by introducing full-length cDNA; UH4 vs the original hTERT1604 (WT) is shown for comparison; error bars are SEM. (c) RT-qPCR showing repression of the ISG *IFI27* in WT10 cells; error bars are not visible for WT10. (d) HT12 array results for UH4 (shown in Figure 2c) versus WT10 confirm most ISG involved in the response pathway are down-regulated again with exception of *BST2*. (e) 450K analysis indicated in contrast that genome-wide methylation (β) levels were largely unchanged from the parental UH4 cells in the WT10 derivatives (f) Methylation across all *HERV* and all *LINE-1* elements assessed by 450K (g) Confirmation of array results using pyrosequencing; there was no significant gain in methylation in WT10 vs UH4 cells; error bars are SD. Figure 5b-c,g: Experiments were performed in triplicate including one biological replicate. Error bars in all experiments represent standard error of the mean (SEM). Significance was denoted as *p < 0.05; **p < 0.01; ***p < 0.001; n.s, non-significant, Student’s T-test.

### Hypomethylated cell lines expressing mutated proteins implicate the Histone 3 tail binding domain of UHRF1 in RE repression

**Figure 6.**
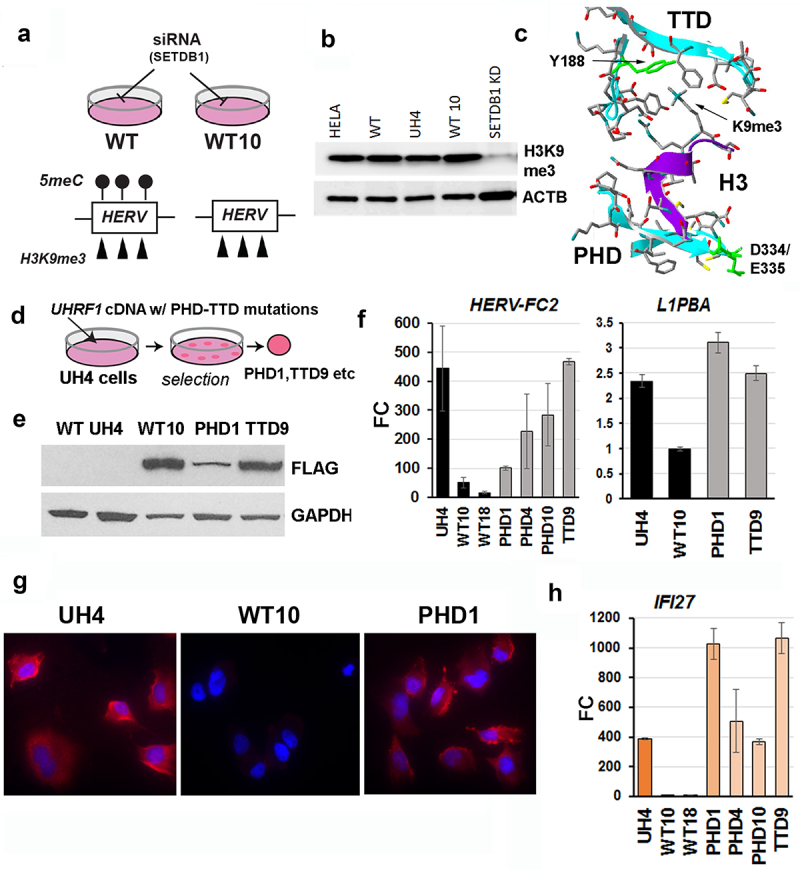
Knockdown cells retained H3K9me3 and could not be rescued with UHRF1 proteins containing H3K9me3 binding site mutations.

We reasoned that H3K9me3, which UHRF1 could recognize with its paired tandem-tudor domain/plant homeodomain (TTD-PHD) [26,27], might be retained by the UHRF1 KD (UH4) cells where there are very low levels of DNA methylation (Figure 6a). Western blotting confirmed that H3K9me3 levels were indeed not substantially decreased in UH4 vs WT cells (Figure 6b): as a control, loss due to transient depletion of the SETDB1 enzyme was readily detected (Figure 6b). The UHRF1 protein engages the histone 3 tail through its TTD-PHD region (Figure 6c), with key residues including D334/E335 (PHD) which holds the tail in place, and Y188 (TTD) which interacts with H3K9me3 [26,27]. We used the same constructs as before to rescue UH4 cells and isolate clones expressing FLAG-tagged UHRF1 proteins containing these mutations in either the TTD (TTD9) or PHD (PHD1, PHD4, PHD10) domains (Figure 6d). These clonal lines showed expression of the FLAG-tagged mutant proteins, with some variation as normally seen in clonally-derived lines (Figure 6e). Unlike cells rescued with intact protein however (WT10, WT18), cell lines containing mutated UHRF1 showed poor and variable repression of RE (Figure 6f) and were positive for dsRNA in the cytoplasm using J2 staining (Figure 6g). In keeping with the failure to repress ERV, IFI27 expression levels remained elevated (PHD1, PHD4, PHD10, TTD9) compared to cells rescued with intact protein (WT10, WT18) (Figure 6h).

(a) Model showing possible differences between original WT cells and WT10 rescues, which may retain H3K9me3 chromatin marks in the absence of DNA methylation; RE are known to have such marks, added by the SETDB1 enzyme (b) Western blot showing levels of H3K9me3 are indeed largely unchanged in hTERT1604 (WT), the UHRF1 KD (UH4) and its rescue line (WT10); actin was used as a loading control (ACTB). Levels in HeLa and SETDB1 KD cells are shown as positive and negative controls, respectively. Four independent siRNAs were used using SMARTpool approach. (c) Model of the paired PHD-TTD domain of UHRF1 interacting with H3K9me3, showing the location of point mutations in the PHD (D334, E335) and TTD (Y188) domains previously shown to affect H3K9me3 binding. (d) Schematic showing approach; UH4 cells were transfected with cDNA as before, but containing the point mutations, and colonies expanded. (e) Example western blot of rescued lines tested for FLAG-tagged proteins. (f) RT-qPCR for individual retrotransposons in the various rescued lines indicated; though variable, repression was generally seen in cells rescued with intact wild-type UHRF1 (WT10, WT18) but not in those containing point mutations in the PHD-TTD region (PHD1, PHD4, PHD10, TTD9); error bars represent SEM. (g) Rescuing UH4 cells with UHRF1 protein caused a shut-down of dsRNA production (WT10) as detected by J2 antibody (red), but not if the protein contained point mutations affecting H3K9me3 binding (PHD1); nuclei were counterstained with DAPI (blue). (h) ISG response to dsRNA is still seen in UH4 cells rescued with point mutated UHRF1 (PHD1, PHD4, PHD10, TTD9), but not in cells rescued with intact protein (WT10, WT18). Figure 6g,h: Experiments were performed in at least duplicate including one biological replicate. Error bars in all experiments represent standard error of the mean (SEM).

## Discussion

We showed here for the first time that depletion of UHRF1 protein in differentiated human cells, either transiently or using stable models, causes loss of DNA methylation, up-regulation of REs and an innate immune response. This was linked to the presence of dsRNA in the cytoplasm, possibly originating from de-repressed REs. Rescuing cells by re-introducing the intact UHRF1 protein caused repression of REs, disappearance of the dsRNA from the cytoplasm, and a switching off of the innate immune response. Notably this rescue effect can occur without reintroducing DNA methylation, suggesting a separate mechanism for RE repression independent of methylated cytosine, but still dependent on UHRF1. Mutations in the PHD/TTD domain prevented rescue and strongly implicated H3K9me3 as the signal which allowed UHRF1 to bind to REs and repress them in demethylated cells. In the absence of ChIP experimentation, we can only allude to this finding.

## The primary cellular response to loss of UHRF1 was RE de-repression and immune activation

The data we present here therefore strongly supports an important role for UHRF1 in suppressing REs and immune activation in human cells. We showed this here using four different approaches: 1)stable KD in normal human lung fibroblasts; 2)transient KD in fibroblasts; 3)transient KD in melanoma cells and 4)bioinformatic analysis of independently published data on transient KD in colon cancer cells. In all cases, we found depletion or mutation of UHRF1 gave up-regulation of REs and induced an innate immune response targeted against dsRNA. Demethylation was seen at most HERV classes examined in our human cell lines, as well as at the more numerous LINE-1 elements in the genome, and we could detect transcriptional activation of several young HERVs and LINE-1 subtypes which have been reported to be recently active and can be de-repressed in response to DNMTi treatment [11,12,15] or loss of H3K9me3 [21].

The de-repression of REs and strong immune response seen here in human, and by others in whole zebrafish [31] and mouse [24] embryos, clearly point to a major role for the protein in maintaining RE suppression. UHRF1 is likely to be most important in differentiated tissues, since *Uhrf1*^*-/-*^ ESC are viable until differentiated *in vitro* [24,25]; likewise, mutations in adult stem cell populations in mouse were not lethal until the cells began to differentiate [33,46]. It is notable that the transcriptional response seen here was dominated by an innate immune activation, indicating that this is the main cellular response to loss of the protein. Responses in cell lines (SKMEL, HCT116) with a more epithelial character also showed a strong but less dominant innate immune response, suggesting that responses may show some variation by cell type, which would be consistent with differences in innate immune signalling abilities [22,47]. However, in all cases RE reactivation and strong innate immune signalling was seen. We see broad activation of RE, yet without direct functional testing, we cannot infer a role for any specific RE in the viral mimicry phenotype [48], which despite a global activation of TEs, only SINE have been found to directly interact with the MDA5 pattern recognition receptor, despite the fact that HERVs were the most upregulated REs, and SINE upregulation was not as marked. While it may or may not be SINE elements in the UHRF1 model, the important take home in this discussion point is that upregulation does not necessarily confer a functional role in viral mimicry, as has been shown before in other models.

## A novel function for UHRF1 in RE repression independent of DNA methylation

Previous work in mouse suggested the ability of UHRF1 to suppress transcription was tightly coupled to its role in assisting the DNA methyltransferases to localize to the nucleus [24,25,29]. In contrast, we showed here that repression could occur without the requirement for DNA methylation. This was shown in i)transient experiments, where endogenous UHRF1 levels were allowed to recover to normal, and ii)in stable KD experiments, where multiple cell lines were derived from UH4 by rescuing with a WT version of the protein (WT10, WT18). In both cases, suppression of both LTR- and non-LTR REs was seen, as well as a switch-off of the innate immune response to viral infection, with the disappearance of dsRNA from the cytoplasm in the case of the stable cell lines. However, neither the transient nor stable cell lines showed any significant restoration of DNA methylation at REs, as assessed using both arrays and pyrosequencing. These results strongly suggest that the presence of UHRF1 alone is sufficient to restore repression, at least in these fibroblast cell lines. These results have three important implications: 1)that DNA methylation in itself is not sufficient to repress REs, at least to a level low enough not to trigger the innate immune response, in these cells; 2)that the UHRF1 protein can mediate repression of the retrotransposons through a mechanism independent of DNA methylation and 3)that there must remain some epigenetic information associated with the RE that allowed UHRF1 to recognize and repress them once protein levels were restored.

## H3k9me3 binding was required for UHRF1-dependent RE suppression

The hypothesis that H3K9me3 binding was required for suppression was strongly supported by introducing point mutations in the H3K9me3 recognition component of UHRF1, which prevented the protein from repressing REs in multiple independent clones. The UHRF1 protein has several functional domains, including the SRA domain, which binds to hemi-methylated DNA [49,50] and the RING and UBL domains, which catalyse the transfer of ubiquitin to histone 3 [51,52]. The failure to repress REs when the PHD or TTD domains were mutated highlighted an essential role for these regions, that cannot be compensated for by other domains. From crystallographic [53] and binding studies [26] it has been shown that the TTD mutation used decreased the protein’s ability to interact with H3K9me3, while the PHD mutations interfered with the protein’s ability to the hold the H3 tail in position and abrogated binding completely [27]. In terms of phenotype and timing, the PHD mutation resembles the *Dnmt1*^*N/N*^ mutation in mice [54] rather than the more severe *Dnmt1*^*S/*S^ or *Dnmt1*^*C/C*^ [55] or *Uhrf1*^*-/-*^ mutants [24,25], all of which died at earlier stages, suggesting we have generated a hypomorphic mutation which decreases rather than abrogates function.

## Conclusions

We have shown here for the first time, using a variety of approaches that UHRF1 is required to suppress RE expression in human cells, including both endogenous retroviruses and LINE-1 elements. Additionally, we have shown that suppression of RE can be achieved by UHRF1 independent of DNA methylation, building evidence for an intricate mechanism for suppression of these elements which warrants further investigation to give insights into the interaction of different RE silencing pathways. This pathway appears to rely on H3K9me3 binding by UHRF1 as mutation of the cognate binding domain on the protein prevents RE suppression. One possibility is that UHRF1 might repress through interactions with the KAP1 corepressor, which has been recently shown to be crucial to RE suppression in human cells [56], but the link between UHRF1 and KAP1 is not yet clear and requires further exploration. The ability of UHRF1 to repress REs without requirement for DNA methylation strongly suggests that this function lies upstream of DNA methylation. Further work is required to determine exactly how UHRF1 can repress these selfish DNA elements and what other components of the cellular machinery are needed for this.

## Supplementary Material

Supplemental Material

## Data Availability

All data have been uploaded to GEO (ncbi.nlm.nih.gov/geo) as GSE128411. All materials and cell lines will be made available from the corresponding author, CPW, upon reasonable request.
