# Supplementary material for: The UHRF1 protein is a key regulator of retrotransposable elements and innate immune response to viral RNA in human cells
Source: Epigenetics. 2023 May 29;18(1):2216005. doi: 10.1080/15592294.2023.2216005 (PMC10228402; doi:10.1080/15592294.2023.2216005)
Supplement: Supplemental Material [file KEPI_A_2216005_SM9005.zip › Supplementary files/Supplementary Materials.docx]

## Supplementary Materials

### Figure S1. Independent UHRF1 KD lines show similar phenotypes

(A) RT-qPCR of *UHRF1* mRNA levels in U5 and U10 cell lines independently derived from hTERT1604 by integration of shRNA; error bars are SEM. (B) Western blot showing levels of UHRF1 protein in U5 and U10; the index cell line UH4 is shown for comparison; GAPDH is a loading control. (C) DNA methylation (β) levels from 450K array analysis of the three *UHRF1* KD lines compared to WT (D) RT-qPCR showing de-repression of the indicated ERV in all three lines; due to high variability not all differences reached significance. (E) Transcription of the indicated ISG genes was significant in all three lines. (F) DNA methylation (β) levels from 450K array analysis of UH4 compared to WT for LINE, LTR and SINE elements (G) RT-qPCR showing lack of de-repression of the indicated SINEs in *UHRF1* KD vs WT. (H) HT12 array results for *DNMT1* levels upon UHRF1 KD; d8, d10, d16 are *DNMT1* KD cell lines. (I) DNA methylation data depicting genomic regions losing methylation in UH4 compared to WT from 450k array. Figure S1A and S1D-E, and F: Experiments were performed in duplicate including one biological replicate. Error bars in all experiments represent standard error of the mean (SEM). Significance was denoted as *p<0.05; **p<0.01; ***p<0.001; n.s, non-significant, Student’s T-test.

### Figure S2. Immunofluorescence staining for all cell types

(A) Immunofluorescence staining for WT, UH4, WT10 and PHD1 cells using antibody against J2 (red) and a DNA counterstain (blue - DAPI), with secondary only controls (AM-IgG; AM – anti-mouse). Representative images from duplicate experiments, taken with ×100 objective on a confocal microscope using identical settings, are shown. (B) Cells in randomly selected microscopic fields from individual repetitions were chosen for fluorescence intensity quantification using ImageJ. The intensity signal was graphed as arbitrary fluorescence units (AU) and were plotted as mean±SD.

**Figure S3. Confirmation of L1 and ERV demethylation and derepression**

(A) RT-qPCR for individual L1 and ERV families indicated; error bars are SEM. (B) Average methylation levels at representative RE as determined by pyroassay; error bars are SEM. (C) DNA methylation levels derived from 450k array probes representative of each repeat element, for WT and UH4.

### Table S1: shRNA sequences

| **Target** | **Supplier** | **Cat. number** | **Mature antisense sequence** | **Cell lines** |
| --- | --- | --- | --- | --- |
| ***UHRF1*** | Dharmacon | V3LHS_ 353720 | TGACATTGCGCACCACCCT | U prefix (U5, U10) |
| ***UHRF1*** | Dharmacon | V3LHS_413692 | AACGTTATATCTTTCTTGG | UH prefix (UH4, UH5) |

### Table S2. siRNA used

| **Target** | **Supplier** | **Cat. number** |
| --- | --- | --- |
| ***UHRF1*** | Horizon Discovery | L-006977-00-0005 |
| ***MAVS*** | Horizon Discovery | L-024237-00-0005 |

All siRNA were ON-TARGETplus Human SMARTpool

### Table S3. Pharmacological Inhibitors

| **Target** | **Supplier** | **Cat. number** |
| --- | --- | --- |
| **Ruxolitinib**  **(INCB018424)** | Selleckchem | S1378 |

### Table S4. Antibodies

| **Target** | **Supplier** | **Cat. number** | **Raised in** | **Clonality** | **Dilution** | **Size (kDa)** | **Application** |
| --- | --- | --- | --- | --- | --- | --- | --- |
| **Primary antibodies** | | | | | | | |
| ***UHRF1*** | SC | 373750 | Mouse | Mono | 1:1000 | 90 | WB |
| ***GAPDH*** | CST | 14C10 | Rabbit | Mono | 1:10000 | 36 | WB |
| **FLAG** | SA | F1804 | Mouse | Mono | 1:1000 | 120 | WB |
| **H3K9ME3** | TFS | # MA5-38430 | Mouse | Mono | 1:1000 | 12 | WB |
| **J2 dsRNA** | SCICONS | 10010200 | Mouse | Mono | 1:500 | X | IF |
| **ACTB** | AB | ab170325 | Mouse | Mono | 1:5000 | 42 | WB |
| **Secondary antibodies** | | | | | | | |
| **AR-IgG** | SC | sc-2004 | Goat | Poly | 1:10000 | X | WB |
| **AM-IgG** | SA | A9044 | Rabbit | Poly | 1:5000 | X | WB |
| **AM-IgG** | Invitrogen | A10036 | Donkey | Mono | 1:1000 | X | IF |

SC (Santa Cruz Biotechnology); CST (Cell Signalling Technologies); SA (Sigma-Aldrich); Thermo Fisher Scientific (TFS); AB (Abcam); Mono (monoclonal); Poly (polyclonal); kDa (Kilodaltons).

### Table S5 Pyrosequencing primers

| **Gene** | **Primer** | **Modification** | **Oligo sequence (5’-3’)** |
| --- | --- | --- | --- |
| ***HERV-FC2*** | FW |  | TTTGGTTTTTTTTGTTAGGATTAGTGA |
|  | RV | biotin | AAATCTCCTCCCCAATTTTAACACCA |
|  | SEQ |  | TTTTTGTTAGGATTAGTGAATT |
| ***HERV-H*** | FW |  | AGGGTTTGTGTGAGTAATAAAGTT |
|  | RV | biotin | ACTCCTACCCCCCAAAAAACAAACT |
|  | SEQ |  | AGTTTTTAATTATTTGGGTGT |
| ***LINE-1*** | FW |  | GGGAGGAGTTAAGATGGT |
|  | RV | biotin | ATAAACCCCATACCTCAA |
|  | SEQ |  | GGGAGGAGTTGGATGGT |

### Table S6: RT-PCR primers

| **Gene** | **Primer** | **Oligo sequence (5’-3’)** |
| --- | --- | --- |
| ***ACT-B*** | FW | GGACTTCGAGCAAGAGATGG |
|  | RV | AGCACTGTGTTGGCGTACAG |
| ***UHRF1*** | FW | TGAGGACATGTGGGATGAGA |
|  | RV | GTCCCTGGAGTTCATCTGGA |

### Table S7: RT-qPCR primers

| **Human RT-qPCR primers** | | |
| --- | --- | --- |
| **Gene** | **Primer** | **Oligo sequence (5’-3’)** |
| ***IFI27*** | FW | TCTGTCCACCCTCTGCTTCT |
|  | RV | GGCATGGTTCTCTTCTCTGC |
| ***OAS2*** | FW | AGGAAGGTAGCGCATCTTGA |
|  | RV | CACCTCTGTCCGACTTGGTT |
| ***STAT1*** | FW | CCCACTCTGATCAACTTTTGC |
|  | RV | GGCCTGTTGAAGATGCTTGT |
| ***ISG15*** | FW | ACCTACGAGGTACGGCTGAC |
|  | RV | GGTGGAGGCCCTTAGCTC |
| ***HERV-FC2*** | FW | TTTCCCACCGCTGGTAATAG |
|  | RV | AGGCTAAGGATTCGGCTGAG |
| ***HERV-H*** | FW | TTCACTCCATCCTTGGCTAT |
|  | RV | CGTCGAGTATCTACGAGCAAT |
| ***HERV-W1*** | FW | AAGAATCCCTAAGCCTAGCTGG |
|  | RV | GCCTAATTAGCATTTTAGTGAGCTC |
| ***L1PBA*** | FW | CTTTGCAGACACTCCCCAGT |
|  | RV | GGTCTAGCCACCCAGCAG |
| ***L1P1*** | FW | TGCCCTAAAAGAGCTCCTGA |
|  | RV | TGTTTTTGCAGTGGCTGGTA |
| ***UHRF1*** | FW | TGAGGACATGTGGGATGAGA |
|  | RV | GTCCCTGGAGTTCATCTGGA |
| ***HPRT*** | FW | AGCCCTGGCGTCGTGATTAGT |
|  | RV | CCCGTTGAGCACACAGAGGCCTA |
| ***EAR*** | FW | GAGGCTGAGGCAGGAGAATCG |
|  | RV | GTCGCCCAGGCTGGAGTG |
| ***ALU*** | FW | CATGGTGAAACCCCGTCTCTA |
|  | RV | GCCTCAGCCTCCCGAGTAG |

### Table S8: sgRNA

| **Human sgRNA oligos** | | | |
| --- | --- | --- | --- |
| **Gene** | **Primer** | **Oligo sequence (5’-3’)** |  |
| ***UHRF1 KO***  ***exon 3 (sg1)*** | FW | CACCGCCATACCCTCTTCGACTACG |  |
|  | RV | AAACCGTAGTCGAAGAGGGTATGGC |  |
| ***UHRF1 KO***  ***exon 3 (sg2)*** | FW | CACCGGCGGACCTCGTAGTCGAAGA |  |
|  | RV | AAACTCTTCGACTACGAGGTCCGCC |  |
| ***UHRF1 PHD mutant***  ***exon 7 (sg1)*** | FW | CACCGGGTCCTGCCGGCCCCCGCAC |  |
|  | RV | AAACGTGCGGGGGCCGGCAGGACCC |  |
| ***UHRF1 PHD mutant***  ***exon 7 (sg2)*** | FW | CACCGCGCACATGAGCTGCTTGTCG |  |
|  | RV | AAACCGACAAGCAGCTCATGTGCGC |  |
| ***UHRF1 PHD mutant***  ***exon 7 (sg3)*** | FW | CACCGCATGTGCGATGAGTGCGACA |  |
|  | RV | AAACTGTCGCACTCATCGCACATGC |  |
