# Supplementary figures and images for: The UHRF1 protein is a key regulator of retrotransposable elements and innate immune response to viral RNA in human cells
Source: Epigenetics. 2023 May 29;18(1):2216005. doi: 10.1080/15592294.2023.2216005 (PMC10228402; doi:10.1080/15592294.2023.2216005)

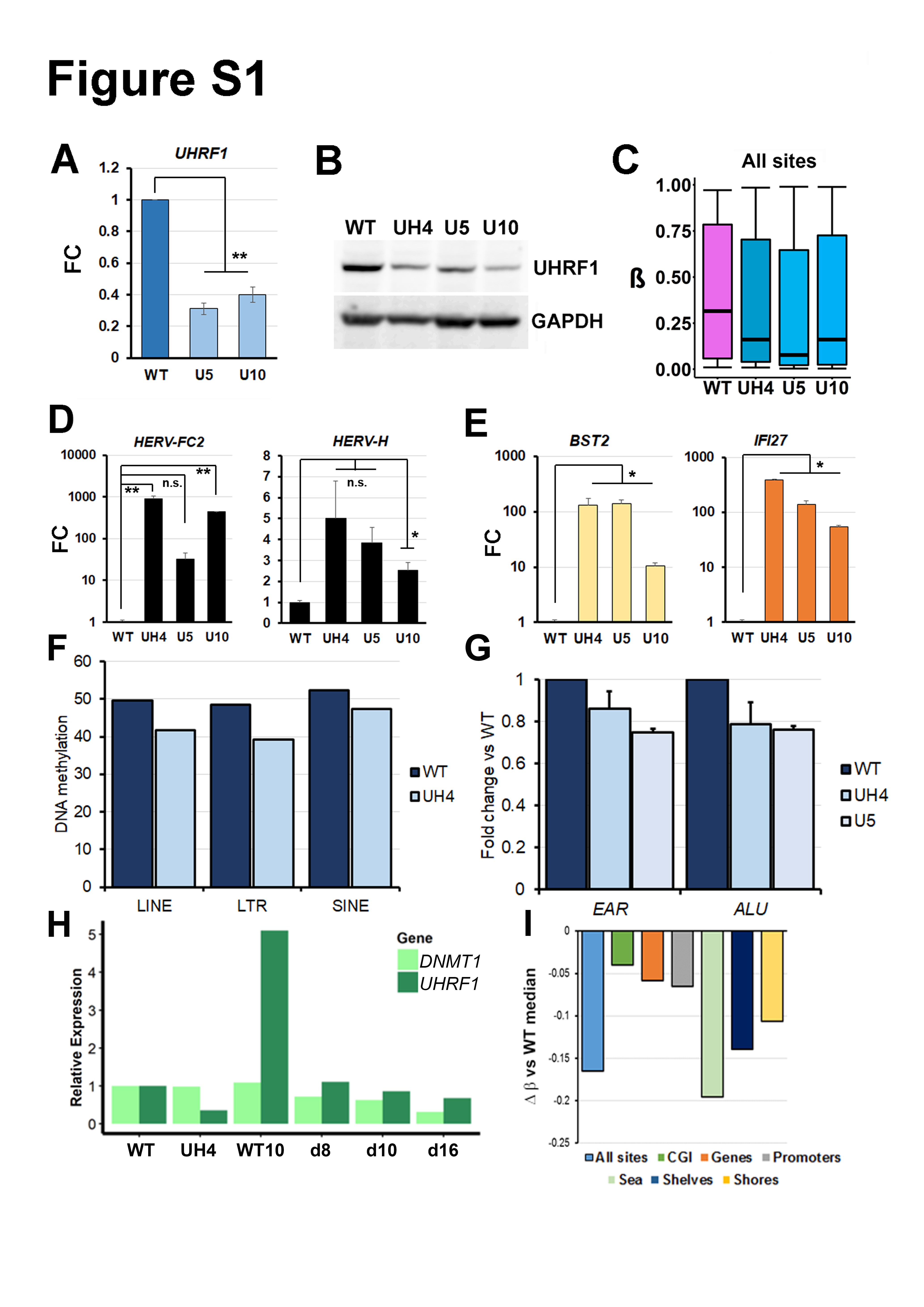

Supplement: Supplemental Material [file KEPI_A_2216005_SM9005.zip › Supplementary files/SUPPLEMENTARY FIGURE 1.tif]

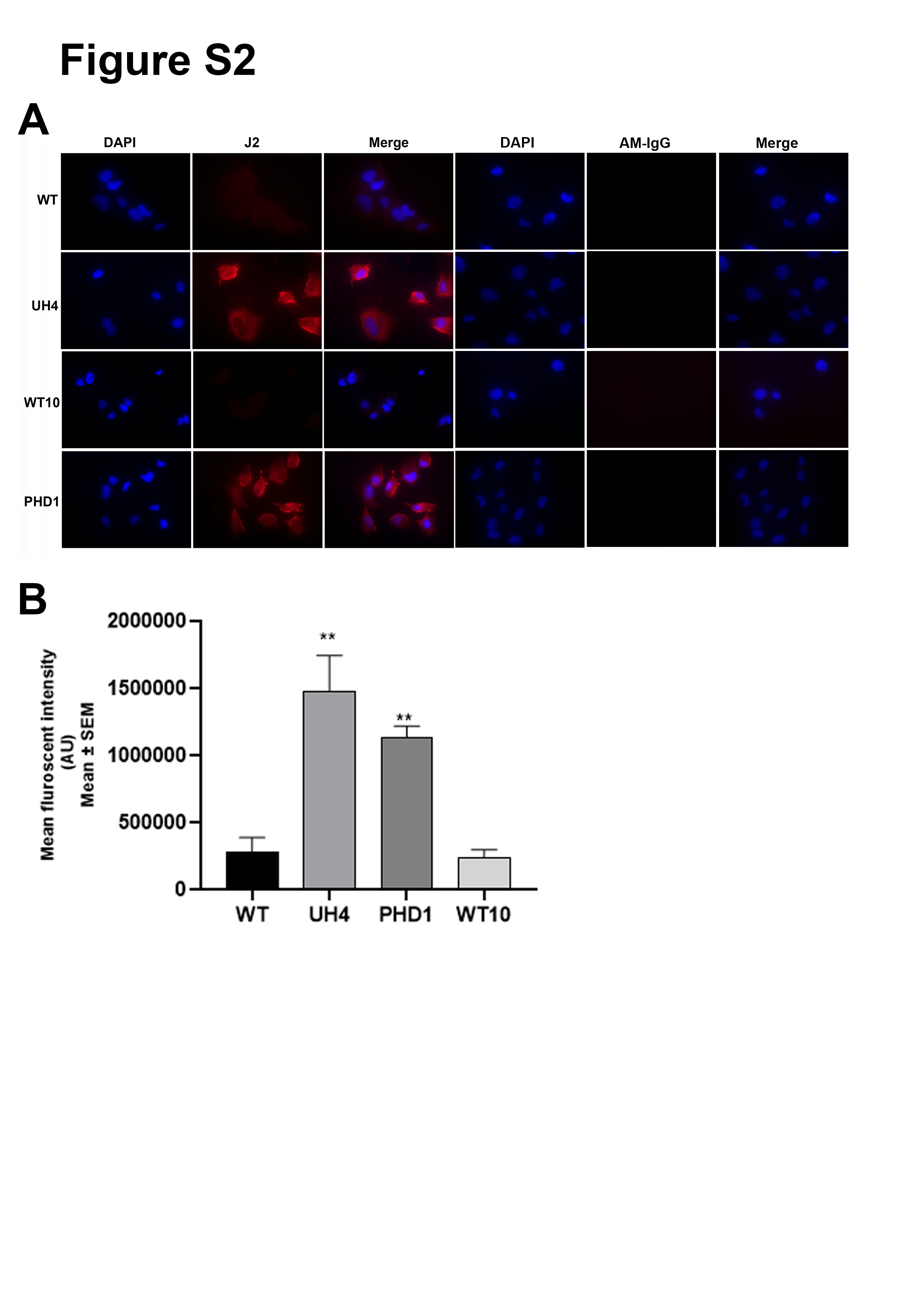

Supplement: Supplemental Material [file KEPI_A_2216005_SM9005.zip › Supplementary files/SUPPLEMENTARY FIGURE 2.tif]

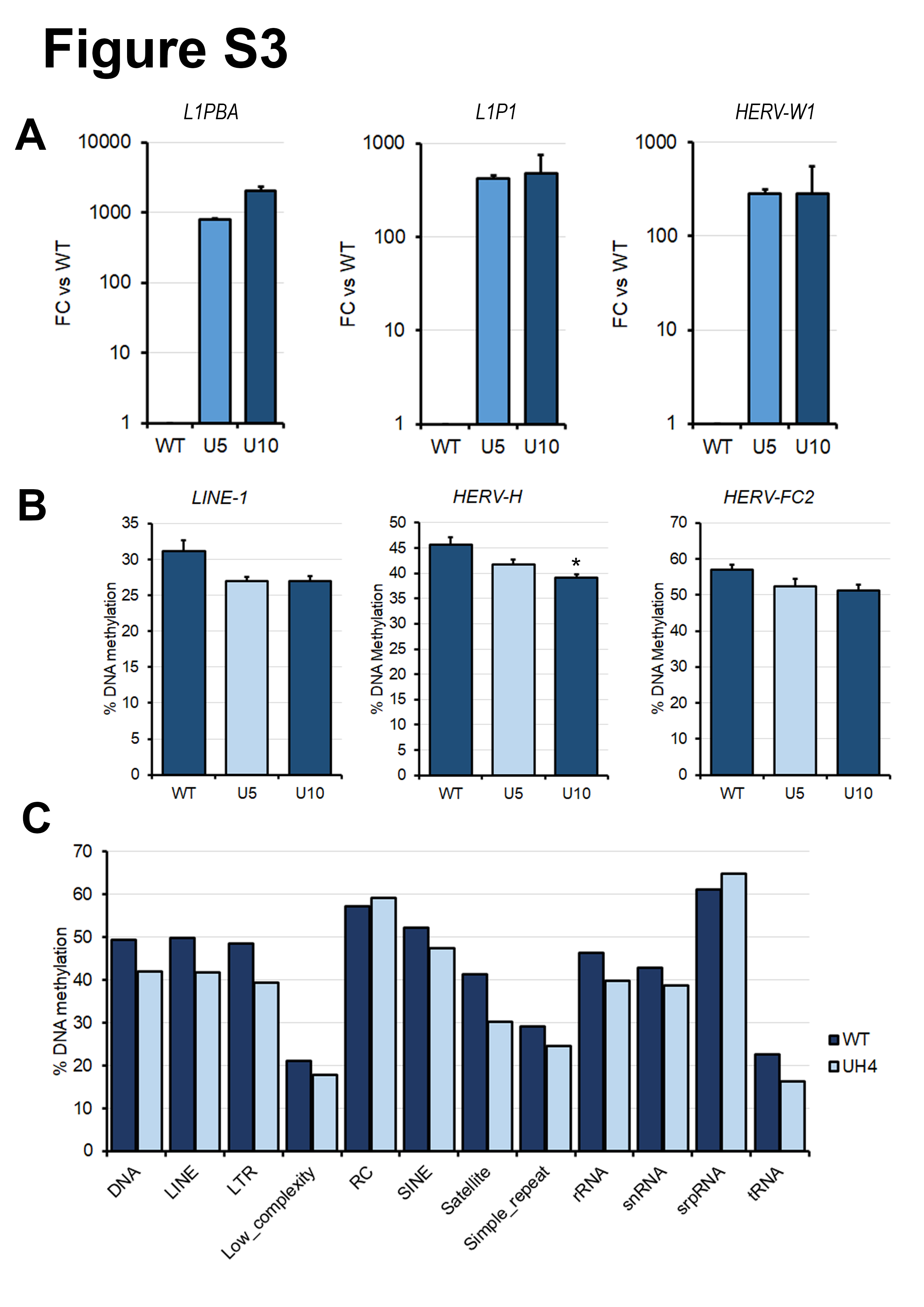

Supplement: Supplemental Material [file KEPI_A_2216005_SM9005.zip › Supplementary files/SUPPLEMENTARY FIGURE 3.tif]
